# Draft Genome Sequence of Pseudomonas abietaniphila KF717 (NBRC 110669), Isolated from Biphenyl-Contaminated Soil in Japan

**DOI:** 10.1128/genomeA.00059-15

**Published:** 2015-03-19

**Authors:** Nobutada Kimura, Atsushi Yamazoe, Akira Hosoyama, Jun Hirose, Takahito Watanabe, Hikaru Suenaga, Hidehiko Fujihara, Taiki Futagami, Masatoshi Goto, Kensuke Furukawa

**Affiliations:** a Bioproduction Research Institute, National Institute of Advanced Industrial Science and Technology (AIST), Tsukuba, Japan; b Biological Resource Center, National Institute of Technology and Evaluation (NITE), Tokyo, Japan; c Faculty of Engineering, Department of Applied Chemistry, Faculty of Engineering, University of Miyazaki, Miyazaki, Japan; d Research Institute for Sustainable Humanosphere, Kyoto University, Kyoto, Japan; e Department of Food and Nutrition, Beppu University, Beppu, Japan; f Faculty of Agriculture, Education and Research Center for Fermentation Studies, Kagoshima University, Kagoshima, Japan; g Faculty of Agriculture, Laboratory of Future Creation Microbiology, Kyushu University, Fukuoka, Japan

## Abstract

Pseudomonas abietaniphila KF717 utilizes biphenyl as a sole source of carbon and energy and degrades polychlorinated biphenyls (PCBs). We report here the 6,930,016-bp genome sequence of this strain, which contains 6,323 predicted coding sequences (CDSs), including the biphenyl-utilizing *bph* gene cluster.

## GENOME ANNOUNCEMENT

Polychlorinated biphenyls (PCBs) have been recognized worldwide as serious environmental pollutants. Pseudomonas abietaniphila strain KF717 was isolated from biphenyl-contaminated soil in Kitakyushu, Japan, as a PCB degrader that utilizes biphenyl as a sole source of carbon and energy (1, 2). Whole-genome shotgun sequencing on strain KF717 was performed by the National Institute of Technology and Evaluation (NITE) by using a combination of shotgun sequencing with a 454 GS FLX+ system (Roche) and paired-end sequencing using a MiSeq sequencing system (Illumina). The Newbler software package (version 2.6; Roche) was used for the genome assembly. The draft genome size is 6,930,016 bp and contains 97 contigs, with an average contig length of 71.4 kb, a median coverage depth of 92-fold, and an average G+C content of 59.4 mol%.

The genome sequence of strain KF717 was uploaded to the RAST server (http://rast.nmpdr.org) to predict the open reading frames (ORFs), tRNAs, and rRNAs (3). Rapid genome annotation using the RAST annotation server revealed 6,323 coding sequences (CDSs), 60 tRNA sequences, and 4 rRNA sequences. The coding sequences were classified into 4,026 subsystems, with the most abundant being systems involved in the metabolism of amino acid derivatives (*n* = 567 CDSs) and carbohydrates (*n* = 509), the production of cofactors, vitamins, prosthetic groups, and pigments (*n* = 301), protein metabolism (*n* = 290), the stress response (*n* = 227), and the production of cell wall and capsule (*n* = 212). A comparison of P. abietaniphila KF717 with other Pseudomonas strains within the RAST database identified Pseudomonas syringae pv. *actinidiae* CFBP 7286 (RAST server genome ID 1094176.3) as its closest neighbor, with a score of 494, followed by P. syringae pv. *phaseolicola* 1448A (genome ID 264730.3), with a score of 481. Pseudomonas fluorescens Q8r1-96 (genome ID 1038923.3) was the sixth closest neighbor, with a score of 459. P. syringae is known as a pathogen of many plant species (4), and P. fluorescens Q8r1-96 was identified as a plant growth-promoting rhizobacterium (PGPR) (5).

The comparison of the functional annotation was performed using the Kyoto Encyclopedia of Genes and Genomes (KEGG) (6). Strain KF717 was found to possess the ring-hydroxylating dioxygenase, the alpha and beta subunits of biphenyl degradation, whole-benzoate degradation via the hydroxylation pathway, and phenol hydroxylase components of the phenol degradation pathway.

### Nucleotide sequence accession numbers.

The whole-genome sequence of P. abietaniphila KF717 has been deposited in DDBJ/EMBL/GenBank under the accession numbers BBQR01000001 to BBQR01000097.
